# The effects of stroboscopic visual training on coordination, change-of-direction, and decision-making performance in collegiate basketball players

**DOI:** 10.3389/fpsyg.2026.1750065

**Published:** 2026-02-18

**Authors:** Yue Li, Shuairan Li, Jielin Yang, Yuerong Hao

**Affiliations:** 1 School of Physical Education and Sports, Shenzhen University of Information Technology, Shenzhen, China; 2 Sports Coaching College, Beijing Sport University, Beijing, China; 3 School of Physical Education, South China University of Technology, Guangzhou, China; 4 School of Physical Education, Qingdao University, Qingdao, China

**Keywords:** basketball-specific training, collegiate athletes, coordination, decision-making performance, reactive agility, stroboscopic visual training

## Abstract

**Objective:**

This study investigated the effects of stroboscopic visual training (SVT) combined with basketball-specific training (ST) on coordination, change-of-direction (COD), and decision-making performance in collegiate basketball players.

**Methods:**

42 male collegiate basketball players (aged 18–25) were classified as Tier 2 (National Level) and randomly assigned to one of three groups: SVT combined with basketball-specific training (SVT + ST, *n* = 14), basketball-specific training (ST, *n* = 14), and regular training (RT, *n* = 14). Intervention effects were evaluated using the Harre Circuit Coordination Test, the 505 Change-of-Direction Speed Test, and a 3D Tactical Animation Decision-Making System. The SVT + ST group performed ST while wearing stroboscopic glasses with progressively increasing difficulty. The ST group underwent identical drills without visual disruption, and the RT group continued with routine basketball training. The intervention lasted 8 weeks, with three 40 min sessions per week.

**Results:**

Significant TIME × GROUP effects were observed. In the coordination test, completion time (*p* = 0.011, ηp^2^ = 0.207) and errors (*p* ≤ 0.01, ηp^2^ = 0.488) improved, with SVT ± ST demonstrating greater gains than ST and RT (completion time: *p* = 0.013, d = 0.763, large; errors vs. RT: *p* ≤ 0.01, d = 4.009). In the 505 COD test, significant effects were identified for completion time (*p* ≤ 0.001, ηp^2^ = 0.69) and asymmetry (*p* ≤ 0.001, ηp^2^ = 0.43), favoring SVT ± ST (completion time vs. ST and RT: *p* ≤ 0.001, d = 3.943, large; asymmetry vs. RT: *p* ≤ 0.001, d = 2.074, large). For decision-making, TIME × GROUP effects were also observed for decision time (DT), motor-execution time (MET), decision accuracy (DA), and the cognitive–motor efficiency index (CMEI) (DT: *p* ≤ 0.001, ηp^2^ = 0.43; MET: *p* ≤ 0.001, ηp^2^ = 0.49; DA: *p* ≤ 0.001, ηp^2^ = 0.48; CMEI: *p* ≤ 0.001, ηp^2^ = 0.79), with SVT ± ST outperforming ST and RT (DT: d = 1.909, large; MET vs. RT: d = 2.102, large; DA: d = 2.341, large; CMEI: d = 3.221, large; all *p* ≤ 0.001).

**Conclusion:**

Integrating SVT with basketball-specific training significantly improved coordination, COD, and decision-making performance in collegiate male basketball players.

## 1 Introduction

Modern basketball is a fast-paced, highly competitive team sport that imposes integrated demands on athletes’ physical, perceptual, and cognitive capacities. During a 40 min elite basketball game, male and female players typically cover ~5–6 km and perform approximately 1,000 discrete actions, requiring speed or direction decisions almost every 2 s. More than 85% of these actions occur above the lactate threshold and at high relative intensities, producing sustained cardiovascular strain with frequent micro-intervals as movement patterns shift every 1–3 s (Stojanović et al., 2018; Dijkstra et al., 2014). In this unpredictable and variable basketball environment, success in training and competition depends not only on traditional loads like running distance or opponent level, but also on the ability to perceive and interpret in real time how situational factors such as close scores, winning streaks and congested schedules affect those loads (Fogt et al., 2021; Sansone et al., 2025). This includes scanning on-court information, recognizing tactical patterns, anticipating opponents, and making adaptive tactical decisions under intense time pressure (Hepler and Feltz, 2012; Ghorbanzadeh et al., 2025). Perceptual–cognitive ability has been identified as a key determinant of both agility and overall game performance (Zemková et al., 2025), with increasing evidence highlighting its role in distinguishing performance levels. In particular, elite athletes consistently outperform sub-elite counterparts in task-specific anticipation and decision-making scenarios (Kioumourtzoglou et al., 1998). As a result, the efficient integration of perceptual–cognitive processing with motor execution has become a decisive factor in competitive basketball, posing unprecedented demands on athletes’ comprehensive performance capacities.

However, although contemporary basketball training increasingly integrates physical, technical, and tactical components, many commonly used practice tasks still isolate specific “physical” or “cognitive” elements to manage coaching constraints and maintain controlled practice conditions. For example, pre-planned change-of-direction (COD) drills can enhance mechanical COD capacity, but because they are executed without an external stimulus, they should be interpreted as COD speed rather than agility (Sheppard and Young, 2006). Accordingly, such drills provide limited opportunities to couple movement execution with rapidly changing opponent- and ball-related visual information under genuine game pressure. Conversely, screen- or video-based perceptual tasks may improve decision accuracy in highly controlled settings, yet their transfer may be limited when perception, decision-making, and whole-body movement must be coordinated simultaneously on court (Nimphius et al., 2018). This practice-to-competition discrepancy reflects an ecological–control trade-off: laboratory-style tasks offer strong control but may under-represent perception–action coupling, whereas representative field tasks improve realism but reduce standardization (Renshaw et al., 2019). This practice-to-competition discrepancy reflects the ecological–control paradox: laboratory-style tasks offer strong experimental control but may under-represent perception–action coupling, whereas representative field tasks improve realism but reduce standardization. SVT can help reconcile this paradox because the stroboscopic eyewear provides a standardized and quantifiable manipulation of visual-information availability (e.g., strobe frequency and duty cycle) that can be applied consistently during on-court, basketball-specific drills. In this way, SVT preserves key sources of ecological validity (whole-body movement, ball–opponent interactions, time pressure, and real-time perception–decision–execution coupling) while retaining experimental controllability over perceptual load and its progression across sessions. This hybrid “controlled perturbation in a representative task” approach may therefore narrow the transfer gap by training players to maintain technical execution and decision quality when visual sampling is intermittently degraded. Therefore, embedding perceptual constraints within basketball-specific drills—such as stroboscopic visual training—may provide a pragmatic compromise to raise perceptual–cognitive demands while preserving representative movement patterns.

To address this gap, perceptual–cognitive training (PCT) has been increasingly used to enhance athletes’ ability to pick up, process, and act on task-relevant information under time pressure. Importantly, basketball-specific evidence supports the relevance and trainability of these skills. For instance, gaze-contingent perceptual training that selectively degraded parts of the visual field improved basketball video-based passing decision accuracy and showed retention effects, suggesting that decision-related information pickup can be trained using sport-specific stimuli (Ryu et al., 2016). In addition, dynamic visual attention measured via multiple-object tracking (MOT) has been linked to basketball match performance, with better tracking performance associated with more assists and steals (Jin et al., 2020), and visual tracking speed has also been associated with productivity indicators in elite (NBA) players (Mangine et al., 2014). Beyond correlational evidence, training studies indicate that MOT-based interventions can improve executive-function outcomes in basketball players (Xiao and Jiang, 2024), supporting the potential for PCT-related constraints to enhance cognitive–perceptual resources relevant to game play. Nevertheless, the ecological–control trade-off remains a central challenge, motivating field-compatible methods that preserve representative movement while systematically manipulating perceptual information.

Stroboscopic visual training (SVT) has emerged as an innovative method in response to the increasing demand for integrated athletic performance (Wilkins and Appelbaum, 2020). By employing stroboscopic glasses that introduce intermittent visual occlusion, SVT artificially induces “visual perturbations” or “stroboscopic load” within the visual channel (Jendrusch et al., 2023). These transient disruptions compel athletes to rely on internal prediction mechanisms, perceptual integration, and multisensory compensation to maintain motor execution. At its core, SVT elevates the processing demands within the perception–action pathway, driving neural adaptations that enhance information extraction and response efficiency under high cognitive load. This mechanism has been associated with improvements in visual sensitivity, reaction speed, and anticipatory ability (Fortes et al., 2025). Empirical studies have shown that SVT enhances visual search efficiency and attentional control and may promote neuroplastic adaptations that support improved motor control and decision-making (Wilkins and Gray, 2015). Promising outcomes have been documented across multiple open-skill sports when SVT is embedded into sport-specific practice tasks. In badminton, stroboscopic interventions have been shown to accelerate visuomotor reaction speed and improve sport-specific defensive performance (e.g., smash-defense), with evidence suggesting that gains may relate to faster visual motion processing rather than purely motor execution changes (Hülsdünker et al., 2019; Hülsdünker et al., 2021b). In volleyball, in-situ stroboscopic training integrated into standard technical drills has produced improvements in complex reaction speed and reactive-agility–related indices, and these changes have been linked to oculomotor adaptations such as altered saccade dynamics (Zwierko et al., 2024; Zwierko M. et al., 2023). In football/soccer contexts, SVT has also been applied within football-specific assessment settings, supporting the idea that intermittent visual occlusion can be implemented without fully removing the representativeness of the task constraints (Palmer et al., 2022).

Despite these potential benefits, the broader SVT literature highlights practical constraints and methodological inconsistencies that limit comparability and translation. Recent systematic reviews and meta-analyses report overall meaningful effects of SVT on both time-based and accuracy-based performance outcomes, while also emphasizing substantial heterogeneity in key prescription variables (e.g., device type, strobe-frequency range, duty-cycle definition and reporting, session duration, and progression rules) and frequent under-reporting of parameters required for reproducibility (e.g., exact Hz settings, duty-cycle progression, and dose-matching rationale) (Jothi et al., 2025; Luo et al., 2025). These reviews further suggest that training efficacy may depend on a “moderate” perturbation window—sufficient to challenge visual sampling and perception–action coupling, but not so severe that it degrades movement quality and alters task solutions—thereby underscoring the need for well-controlled, dose-matched trials that specify and justify strobe parameters within representative sport-specific drills (Wang Y. et al., 2025).

SVT may also help address the ecological control paradox in basketball because stroboscopic eyewear enables standardized, quantifiable manipulation of visual information availability (e.g., strobe frequency and duty cycle) that can be implemented consistently during on-court, basketball-specific drills (Wilkins and Gray, 2015). In this way, SVT can preserve key elements of ecological validity—including whole-body movement, ball–opponent interactions, time pressure, and real-time coupling of perception, decision-making, and action execution—while retaining experimental control over perceptual load and its phase-based progression (Ren et al., 2025). This hybrid approach, involving controlled perturbation within representative tasks, may train players to maintain technical execution and decision quality when visual sampling is intermittently disrupted, thereby narrowing the transfer gap (Wang R. et al., 2025). Given that basketball performance requires rapid visual sampling, continuous updating of opponent–ball information, and time-limited perception–action coupling, embedding stroboscopic perturbations within representative basketball drills may help athletes sustain decision quality and movement execution when visual information is degraded (Guo et al., 2025). Accordingly, a basketball-specific randomized trial that dose-matches sport-specific training content while manipulating only visual perturbation is warranted to clarify efficacy and inform practicable standardization (Zhao et al., 2025).

Accordingly, this study aims to systematically investigate the effects of combining SVT with sport-specific training on coordination, agility, and decision-making performance in collegiate basketball players. Through an eight-week randomized controlled trial, the study seeks to determine whether SVT can yield greater performance enhancements in competition-relevant tasks compared to conventional training methods. The findings are expected to elucidate the mechanisms by which visual perturbation influences the perception–action coupling process and to provide empirical evidence for developing standardized SVT protocols and multidimensional performance assessment frameworks. Ultimately, this study intends to offer both theoretical insights and practical guidance for optimizing integrated training strategies in basketball and other team sports.

## 2 Methods

### 2.1 Participants

The required sample size was estimated using G*Power software (version 3.1.9.7; Franz Faul, Kiel University, Germany). The parameters were set as follows: significance level (*α*) = 0.05, statistical power (1 − *β*) = 0.80, and effect size (*f*) = 0.40. A repeated-measures ANOVA with an *F*-test for the group × time interaction was selected. The analysis yielded a minimum required sample size of 24. Accounting for an anticipated attrition rate of approximately 20% and a three-group design (SVT + ST, ST, and RT), a total of 45 participants were recruited (*n* = 14 per group). Three participants withdrew during the intervention because of competition commitments, and 42 participants ultimately completed the full intervention protocol. Participants were recruited between March 2 and March 27, 2025, and the formal intervention phase was conducted from April 1 to May 12, 2025. Participant performance caliber was classified using the Participant Classification Framework proposed by McKay et al. (2022). Based on (i) ≥ 5 consecutive years of basketball-specific training, (ii) current enrollment as collegiate team players, and (iii) participation in ≥50% of official collegiate games during the previous season, the present cohort was categorized as Tier 2 (National Level). All participants were male collegiate basketball players enrolled at several local universities, and met the following inclusion criteria: (1) male; (2) aged 18–25 years; (3) a minimum of five consecutive years of basketball-specific training experience; (4) participation in at least 50% of official collegiate games during the previous season; and (5) normal or corrected visual acuity of 1.0 or above. Exclusion criteria were as follows: (1) history of concussion, vestibular dysfunction, or ankle/lower limb injury within the past 3 months; (2) any clinical condition potentially affecting balance or sensorimotor adaptation; and (3) concurrent involvement in other visual training programs. The study complied with the ethical principles outlined in the Declaration of Helsinki and was approved by the Ethics Committee of Sports Science Experiments at Beijing Sport University (Approval No. 2024-BSU-164H). All participants received detailed information regarding the study procedures and provided written informed consent prior to participation. Detailed participant characteristics are presented in Table 1.

**Table 1:** Characteristics of participants.

Measure	SVT + ST (*n* = 14)	ST (*n* = 14)	BC (*n* = 14)	*p*-value
Age (years)	20.1 ± 1.2	20.3 ± 1.5	20.5 ± 1.8	0.79
Height (cm)	183.4 ± 3.0	182.6 ± 5.1	186.9 ± 6.2	0.06
Mass (kg)	75.5 ± 8.3	74.3 ± 6.4	79.2 ± 6.0	0.17
BMI (kg/m^2^)	23.0 ± 1.2	23.0 ± 1.9	23.6 ± 1.2	0.47

### 2.2 Study design

**Figure 1:**
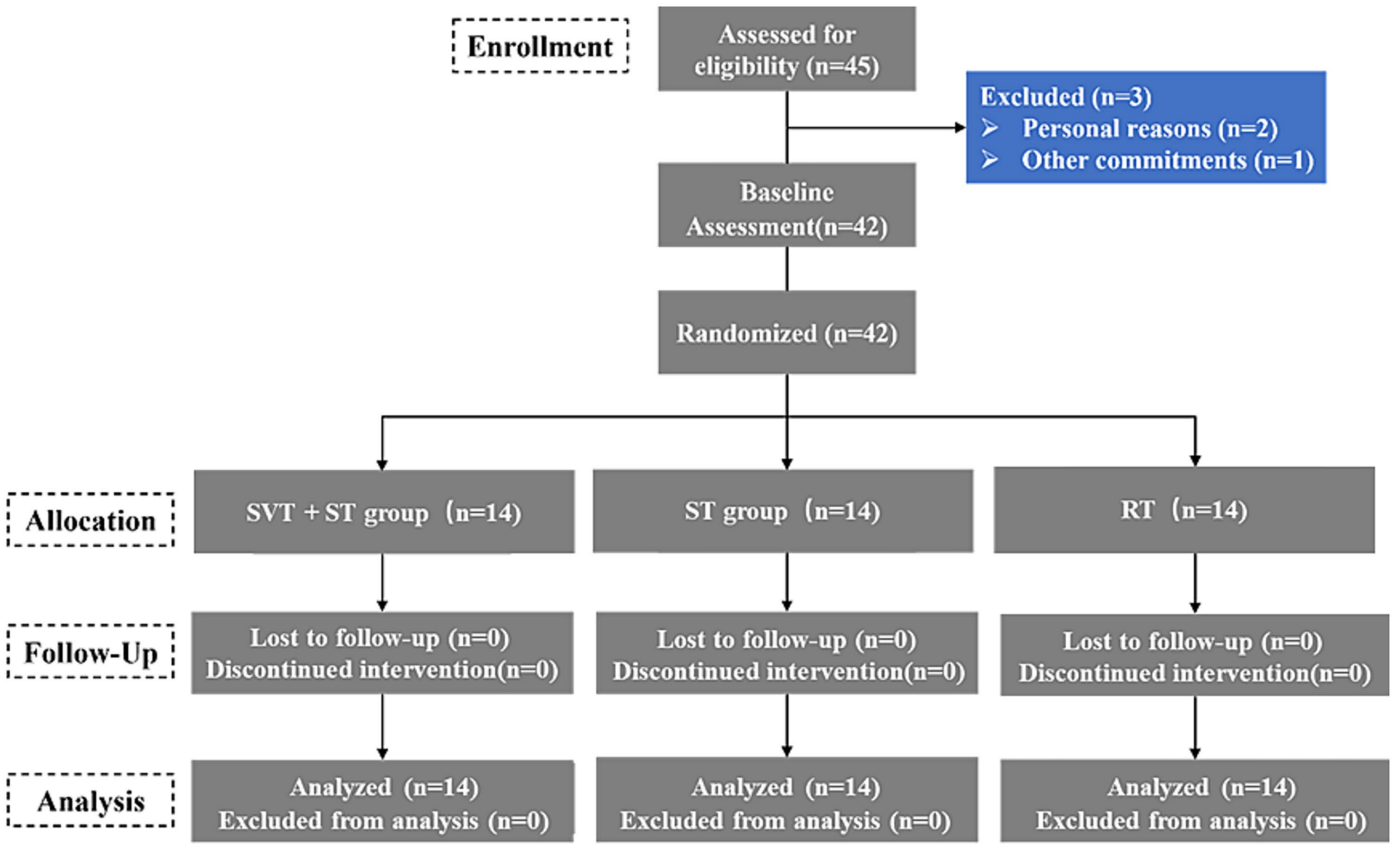
CONSORT flow diagram of participants through the trial.

As illustrated in Figure 1, this study employed a three-arm, parallel-group, assessor-blinded randomized controlled trial design. Forty-two participants were randomly assigned in a 1:1:1 ratio, An independent statistician generated the allocation sequence in a 1:1:1 ratio using SAS 9.4 with permuted blocks (block size = 6) and uploaded it to the REDCap centralized randomization module. Allocation was concealed until completion of all baseline assessments; group assignments were released automatically by the system thereafter. Participant blinding was not feasible because the use of stroboscopic eyewear is perceptible to participants; however, participants were not informed of the study hypotheses/expected direction of effects and were instructed not to disclose their training condition to the assessors during testing. Using this procedure, 42 participants were randomized to: (1) stroboscopic visual training combined with sport-specific training (SVT + ST, *n* = 14); (2) sport-specific training alone (ST, *n* = 14); and (3) regular training (RT, *n* = 14). During basketball-specific training, participants in the SVT + ST group wore progressive stroboscopic glasses (Nike Vapor Strobe Eyewear, Nike Inc., Beaverton, OR, United States), with the strobe frequency progressively reduced from 15 Hz to 9 Hz and duty cycle progressively increased across phases (50% → 50–60% → 60–70%). The ST group performed the same basketball-specific drills without visual occlusion, while the RT group participated in standard team basketball practice with no additional intervention. All performance assessments were conducted by independent assessors who were blinded to group allocation. Data were analyzed using coded group labels by an independent statistician to further minimize bias. The intervention lasted 8 weeks and included three sessions per week, each approximately 40 min in duration. The SVT + ST and ST groups received identical training content, frequency, and workload, differing only in the presence or absence of stroboscopic stimulation. To ensure adherence and monitor participant safety, session attendance was recorded, with a minimum compliance threshold set at 85% (i.e., at least 20 out of 24 sessions completed). Any adverse symptoms reported during training, such as dizziness, visual fatigue, or nausea, were monitored and documented throughout the intervention period.

### 2.3 Training intervention

The intervention incorporated stroboscopic visual training (SVT) using stroboscopic eyewear (Nike Vapor Strobe Eyewear, Nike Inc., Beaverton, OR, United States), which was programmed and controlled via Bluetooth through the Senaptec Strobe smartphone application. SVT was embedded within skill training (ST) to increase perceptual–cognitive challenge while preserving representative basketball movement patterns and perception–action coupling (Appelbaum and Erickson, 2018; Fransen et al., 2017). The standardized 40 min intervention blocks for the SVT ± ST and ST groups were delivered within the teams’ regular training time (Monday/Wednesday/Friday) and replaced an equivalent duration of routine training (RT). Therefore, total weekly training frequency and duration were comparable across groups, and differences can be attributed primarily to the presence (SVT ± ST) versus absence (ST) of stroboscopic visual occlusion rather than additional training volume.

Program development was informed by field-based SVT/restricted-vision practice in open-skill sports, where intermittent visual occlusion is integrated into sport-specific drills and progressively intensified to overload visuomotor processing without abandoning task representativeness (Appelbaum and Erickson, 2018; Wilkins and Appelbaum, 2020). Specifically, we adapted progression principles and implementation logic from stroboscopic and restricted-vision training reported in soccer/football (e.g., restricted-vision constraints applied during ball-control tasks), volleyball (in-situ stroboscopic training embedded in sport-specific tasks), and badminton (elite-level, sport-specific SVT improving visuomotor performance), and then tailored drill ecology and progression targets to the unique constraints of basketball (Ren et al., 2025; Jothi et al., 2025; Wang Y. et al., 2025).

Basketball requires frequent, time-limited visual sampling and rapid updating of action choices while the body is moving (e.g., dribbling under pressure, reading closeouts, identifying passing windows, coordinating footwork for stop–go transitions, and executing shots under defensive proximity). Therefore, the intervention was structured to (i) maintain basketball-specific information sources (opponent/ball/space cues and coach-defined tactical cues), (ii) preserve representative actions (ball-handling, passing, shooting, COD footwork, and small-sided decision contexts), and (iii) progressively increase visual uncertainty so athletes must rely more on predictive control, peripheral monitoring, and efficient cue use—consistent with practitioner-oriented SVT recommendations emphasizing representative tasks and progressive occlusion loading for feasible on-court implementation (Appelbaum and Erickson, 2018; Dingirdan Gultekinler et al., 2025).

The intervention lasted 8 weeks, with three supervised sessions per week (Monday/Wednesday/Friday) and ~40 min per session. To ensure internal validity, the SVT ± ST and ST groups were dose-matched for session frequency, total duration, drill content, work: rest structure, and coaching instructions; the only difference was the presence (SVT ± ST) versus absence (ST) of stroboscopic occlusion. The RT group continued routine team training without the standardized intervention blocks. Progression followed a four-stage model consistent with motor-learning and overload principles, moving from stable technique under mild perturbation to high-speed, game-representative decisions under stronger perturbation. Visual perturbation was increased primarily by reducing strobe frequency (less continuous visual information per unit time) and, in later stages, by increasing duty cycle within tolerable ranges. This staged approach reflects evidence and practical guidance indicating that gradually increasing occlusion load supports tolerability and preserves movement quality compared with maximal occlusion from the outset.

Stage 1 (Weeks 1–2) prioritized foundational ball-handling/passing and set-shot routines combined with simple cue–response decisions to stabilize technique while athletes adapt to intermittent visual information. Stage 2 (Weeks 3–4) increased locomotor demands (e.g., moving shots, COD dribble routes) and introduced multi-choice decisions to strengthen perception–action coupling while changing direction and controlling the ball. Stage 3 (Weeks 5–6) emphasized higher-speed execution and dual-task constraints (e.g., peripheral cue detection while dribbling/shuttling) and incorporated structured 2v1/3v2 scenarios to train reading and acting under time pressure. Stage 4 (Weeks 7–8) maximized representativeness using small-sided competitive segments (e.g., 3v3/4v4) and random tactical commands to train rapid cue pickup, action selection, and team coordination under degraded visual sampling. For safety and fidelity, all sessions were supervised, attendance was recorded, and participants reported adverse symptoms (e.g., dizziness, visual fatigue). Strobe parameters were fine-tuned only within pre-specified stage ranges when needed to maintain tolerability and task completion quality, while preserving dose-matching between SVT ± ST and ST. Full details of the training intervention, parameter specifications, and a representative weekly training summary are presented in Appendix 1.

### 2.4 Measurements

All assessments were conducted twice: once prior to the intervention (baseline) and once after the 8-week training program. To minimize potential learning effects, all participants completed a standardized familiarization session 1 week before the pre-test, during which each assessment was practiced 2–3 times. The testing sequence was standardized as follows: decision-making test → coordination test → 505 change-of-direction speed test. Cognitive assessments were administered first to avoid the confounding effects of physical fatigue on decision-making performance. A minimum rest period of 10 min was provided between consecutive tests. All assessments were administered by independent evaluators who were trained in standardized protocols and remained fully blinded to participants’ group allocation throughout the data collection process.

#### 2.4.1 Coordination test

Whole-body coordination was assessed using a modified version of the Harre Circuit Test. Originally developed by German sports scientist Dietrich Harre to evaluate overall coordination, the test has been widely adopted across various sports disciplines (Ljach and Witkowski, 2010). In the present study, we adapted the initial movement of the original protocol (forward roll) to a basketball-specific defensive slide and pivot sequence to enhance task specificity while preserving the construct of “whole-body coordination” (i.e., rapid sequencing of multidirectional movements, spatial orientation, and body-control demands) (Scanlan et al., 2014). It has also been implemented as a general motor-coordination outcome in youth sport settings, with high test–retest reliability reported when timing is captured using photocell systems (e.g., ICC > 0.95 in preadolescent soccer players) (Trecroci et al., 2015). The testing area consisted of a 6 m × 5 m rectangular field containing one marker pole, three hurdles (each 75 cm high and spaced 2.5 m apart), and a 1 m × 1 m foam mat. Participants began in a defensive slide stance behind the starting line and, upon command, executed a 3 m defensive slide followed by a 180° pivot and sprint. They then completed a hurdle-over–under sequence and concluded the test by leaping over the mat and crossing the finish line. Both completion time and technical errors were recorded. Each participant performed three trials, with the fastest valid trial used for analysis. Completion time was measured using an automatic photoelectric timing system, while trained evaluators simultaneously documented technical errors, including foot crossover, hurdle contact, or incomplete pivots. The basketball-adapted Harre Circuit Test demonstrated enhanced sport specificity without compromising the validity of coordination assessment. Pilot testing confirmed its reliability, with an intraclass correlation coefficient (ICC) of 0.89 and inter-rater agreement (Kappa = 0.88), supporting its robustness and practical applicability for evaluating coordination in basketball settings. Detailed testing procedures are provided in Appendix 2.

#### 2.4.2 505 change-of-direction speed test

The 505 Change-of-Direction Speed Test was employed to evaluate participants’ ability to decelerate, execute a 180° change of direction, and reaccelerate under high-speed movement conditions (Draper and Lancaster, 1985). Developed by the Australian Institute of Sport, this standardized protocol has been widely utilized in basketball research and exhibits excellent test–retest reliability (ICC = 0.94–0.96) and strong criterion validity (Nimphius et al., 2018). The test was conducted on a 15 m straight sprint lane, with a photocell timing gate positioned 5 m before the turning line. Participants performed a flying start from the baseline, sprinted through the timing gate toward the turning line, touched the line, rapidly executed a 180° turn, and sprinted back through the gate to stop the timer. The effective timed distance was 10 m (5 m entry + 5 m return). Each participant completed three trials on both the dominant and non-dominant leg as the plant leg, and the fastest valid trial for each was recorded. An asymmetry index (AI) was calculated, with values exceeding 10% interpreted as indicative of functional lower-limb asymmetry. An asymmetry index (AI) was calculated. Following common practice in the inter-limb asymmetry literature, a threshold around 10% is often used as a pragmatic flag for potentially meaningful between-limb differences in applied sport testing; however, such thresholds are task-, metric-, and population-specific and should not be interpreted as universal diagnostic cut-offs (Bishop, 2021; Parkinson et al., 2021). For the 505 test, the smallest detectable difference has been reported to be approximately 3–4%, suggesting that a 10% discrepancy typically exceeds measurement error and can be used as a conservative descriptive indicator (Barber et al., 2016). Accordingly, we interpreted AI ≥ 10% as a monitoring threshold and also reported AI as a continuous variable, because COD asymmetry may reflect direction/limb-specific braking–propulsion strategies and technical preferences during 180° turns (Dos' Santos et al., 2019; Thomas et al., 2020). The 505 change-of-direction Test is considered a valid and reliable measure of short-distance acceleration and directional control and is particularly suited for sports involving frequent directional changes, such as basketball and rugby. In basketball-specific populations, it has demonstrated high reliability (ICC = 0.91–0.93), along with strong validity and reproducibility. Detailed testing procedures are described in Appendix 3.


$$\mathrm{AI}=\frac{|T_{\mathrm{dom}}-T_{\text{non}}|}{\frac{T_{\mathrm{dom}}+T_{\text{non}}}{2}}\times 100$$

Note: *T_dom_* = completion time for the dominant leg (s); *T_non_* = completion time for the non-dominant leg (s).

#### 2.4.3 Decision-making test

A customized decision-making assessment system integrating three-dimensional (3D) tactical animations with immediate real-action execution was used to evaluate perception-judgment-action performance under simulated basketball conditions. The 3D scenarios were developed in Unity and presented via E-Prime 3.0 on a 65-inch 4 K screen (participants standing 3 m from the display). Thirty representative clips (3–5 s each; 10 fast-break 2v1, 10 pick-and-roll 3v2, and 10 corner-spacing situations) were derived from match footage and validated by an expert panel (content validity index, CVI = 0.91). When the animation reached the pre-defined decision frame, the video froze for 0.5 s to provide a stable decision cue. DT was defined as the latency from freeze onset (t = 0 ms) to the key press. Participants were required to respond within 800 ms from freeze onset; if no response occurred during the 0.5 s freeze, the video resumed while the 800-ms response window remained anchored to freeze onset. Accordingly, the freeze was implemented to standardize cue exposure rather than to prolong DT, which was consistently time-locked to freeze onset across all trials. Decision time (DT, ms) was defined as the interval from freeze onset to key press, and decision accuracy (DA%) was calculated as the percentage of correct decisions relative to the expert-consensus solution. Immediately after the on-screen feedback, participants executed the corresponding basketball action (pass to a 1 m x 1 m target at 5 m; three-point shot from 6.75 m; or a 5 m drive to a cone and return). Movement execution time (MET, ms) was predefined as the interval from feedback onset to movement completion. Movement execution time (MET) was used to quantify the duration of the action phase within the perception, decision, and action chain. In this study, MET was operationally defined as the interval from the onset of an execution cue presented after participants confirmed their decision via a button press to the occurrence of the movement completion event, rather than being simply defined as the interval from button press to movement completion. Specifically, immediately after pressing the button, participants performed the corresponding basketball action: (1) pass, with movement completion defined as ball release; (2) shot, with movement completion defined as ball release; and (3) drive, with movement completion defined as completion of a prescribed 5 m drive and return task (i.e., returning to the endpoint marker after touching the target marker). MET (ms) was determined by frame-by-frame coding from synchronized high-speed video (240 fps) and converted to milliseconds using the frame count (MET = frames × 1000/240). Accordingly, MET reflects actual movement execution duration under standardized task constraints. No inertial sensors were used, and MET was not inferred from button timing, thereby avoiding conflation of cognitive reaction time with movement duration.

For passing and shooting, completion was defined as ball release, whereas for driving it was defined as returning to the endpoint. MET was obtained from synchronized high-speed recordings using a high-speed camera (Sony RX100 VII, 240 fps) together with an eye-tracking system (Insight V3.0), rather than being inferred from button presses. The cognitive-motor efficiency index (CMEI) was computed as CMEI = DA%/ (DT + MET). Three practice trials were provided before formal testing to minimize familiarization effects, and the 30 scenarios were presented in randomized order with standardized rest periods. The 30-scenario test battery demonstrated good internal consistency (Cronbach’s alpha = 0.82) and criterion-related validity, showing significant correlations with assist-to-turnover ratio and coach performance ratings (r = 0.58–0.64, *p* < 0.01). Detailed testing procedures are provided in Appendix 4.


$$\text{CMEI}=\frac{\mathrm{DA}\%}{\mathrm{DT}+\mathrm{MET}}$$

### 2.5 Statistical analysis

All statistical analyses were performed using JASP software (version 0.18.3; JASP Team, The Netherlands). Results are reported as mean ± standard deviation (SD). The Shapiro–Wilk test was used to assess the normality of each variable. For data meeting the normality assumption, one-way analysis of variance (ANOVA) was conducted to examine between-group differences at baseline for demographic variables (age, height, weight, BMI) and key outcome measures, including Completion Time (s), Error Count (*n*), Left–Right Difference (%), Decision Time (DT, ms), Motor Execution Time (MET, ms), Decision Accuracy (DA%), and the Cognitive–Motor Efficiency Index (CMEI). To evaluate intervention effects, a two-way repeated-measures ANOVA (group × time) was performed, incorporating the between-subjects factor group (SVT + ST, ST, RT), the within-subjects factor time (pre-intervention, post-intervention), and their interaction. When significant interaction effects were detected, Bonferroni-adjusted *post hoc* comparisons were conducted to identify the source of differences. Statistical significance was set at *p* < 0.05, and all tests were two-tailed. Effect sizes were calculated using Cohen’s d for within-group comparisons and partial eta squared (ηp^2^) for between-group effects. Interpretation of Cohen’s d followed the thresholds: small (0.2), moderate (0.5), large (0.8). For ηp^2^, effect sizes were categorized as small (0.01), moderate (0.06), and large (0.14) (Cohen, 2013).

## 3 Results

**Figure 2:**
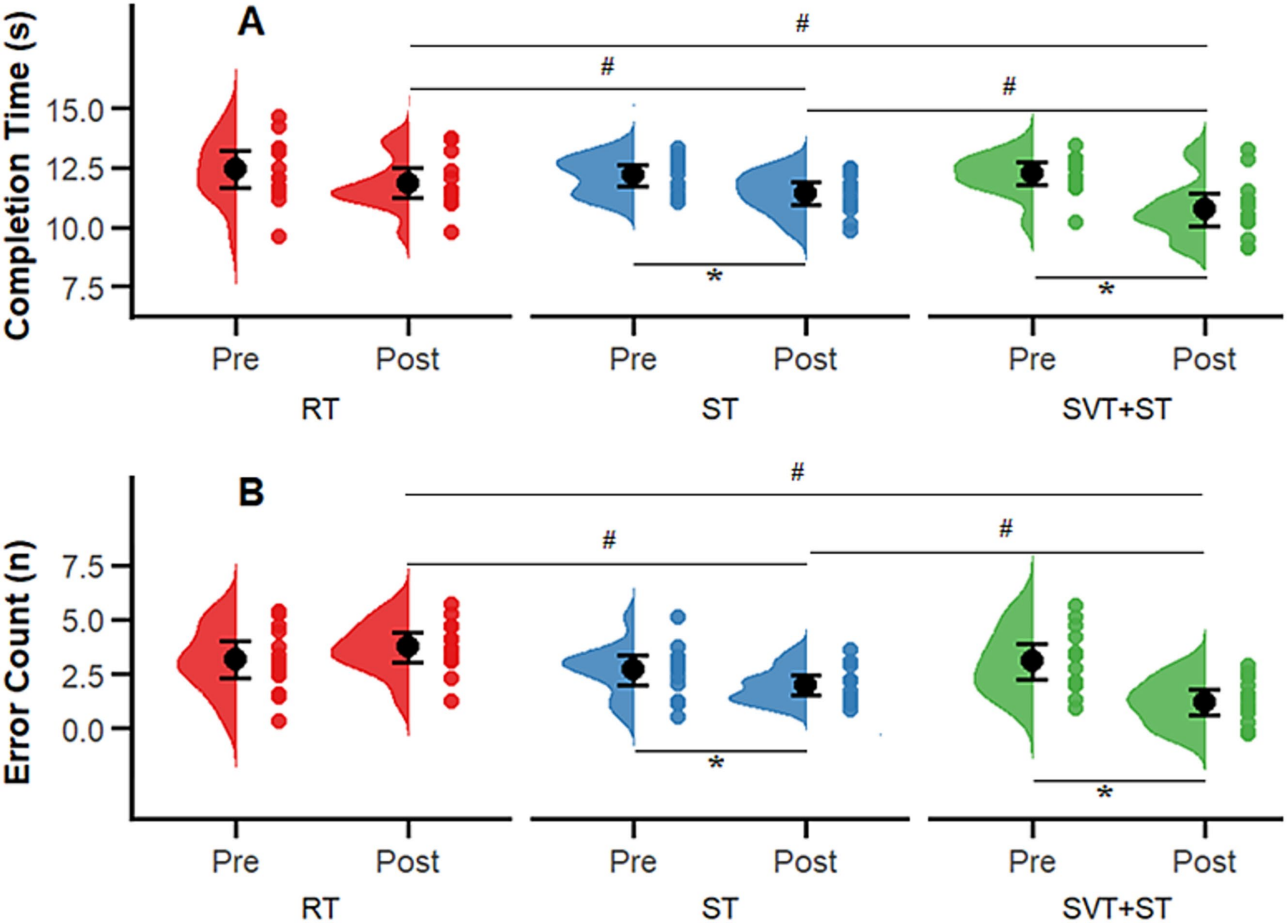
Comparison of Harre circuit coordination test results before and after the 8-week intervention across the SVT + ST, ST, and RT groups. **(A)** completion time (s); **(B)** error count (n). Distributions and individual data points are shown for Pre and Post; black dots with error bars indicate the group mean and its uncertainty. * indicates a significant within-group Pre–Post difference (*p* < 0.05); # indicates a significant between-group difference (*p* < 0.05).

**Figure 3:**
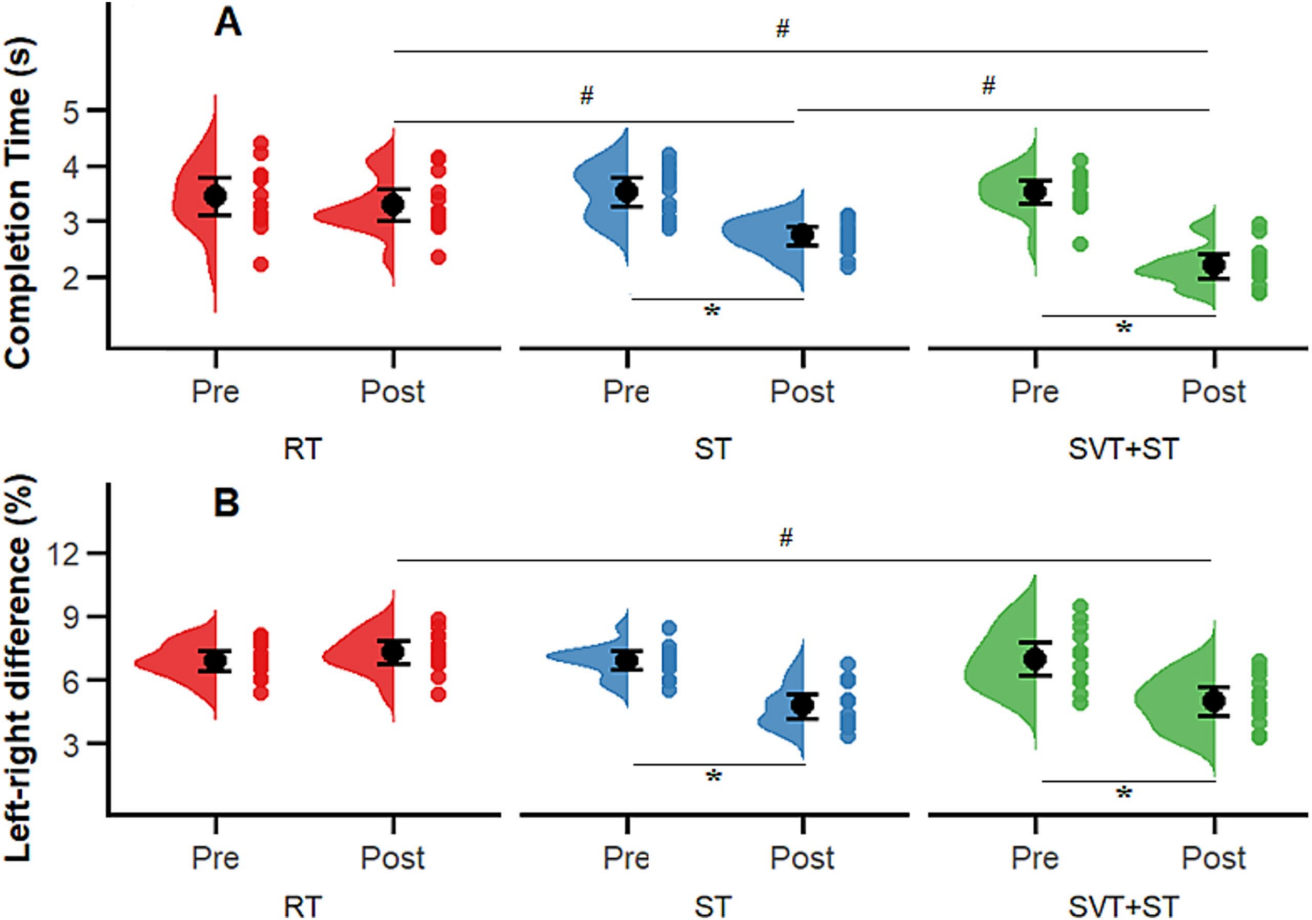
Comparison of 505 agility test results before and after the 8-week intervention across the SVT + ST, ST, and RT groups. **(A)** completion time (s); **(B)** error count (n). Distributions and individual data points are shown for Pre and Post; black dots with error bars indicate the group mean and its uncertainty. * indicates a significant within-group Pre–Post difference (*p* < 0.05); # indicates a significant between-group difference (*p* < 0.05).

**Figure 4:**
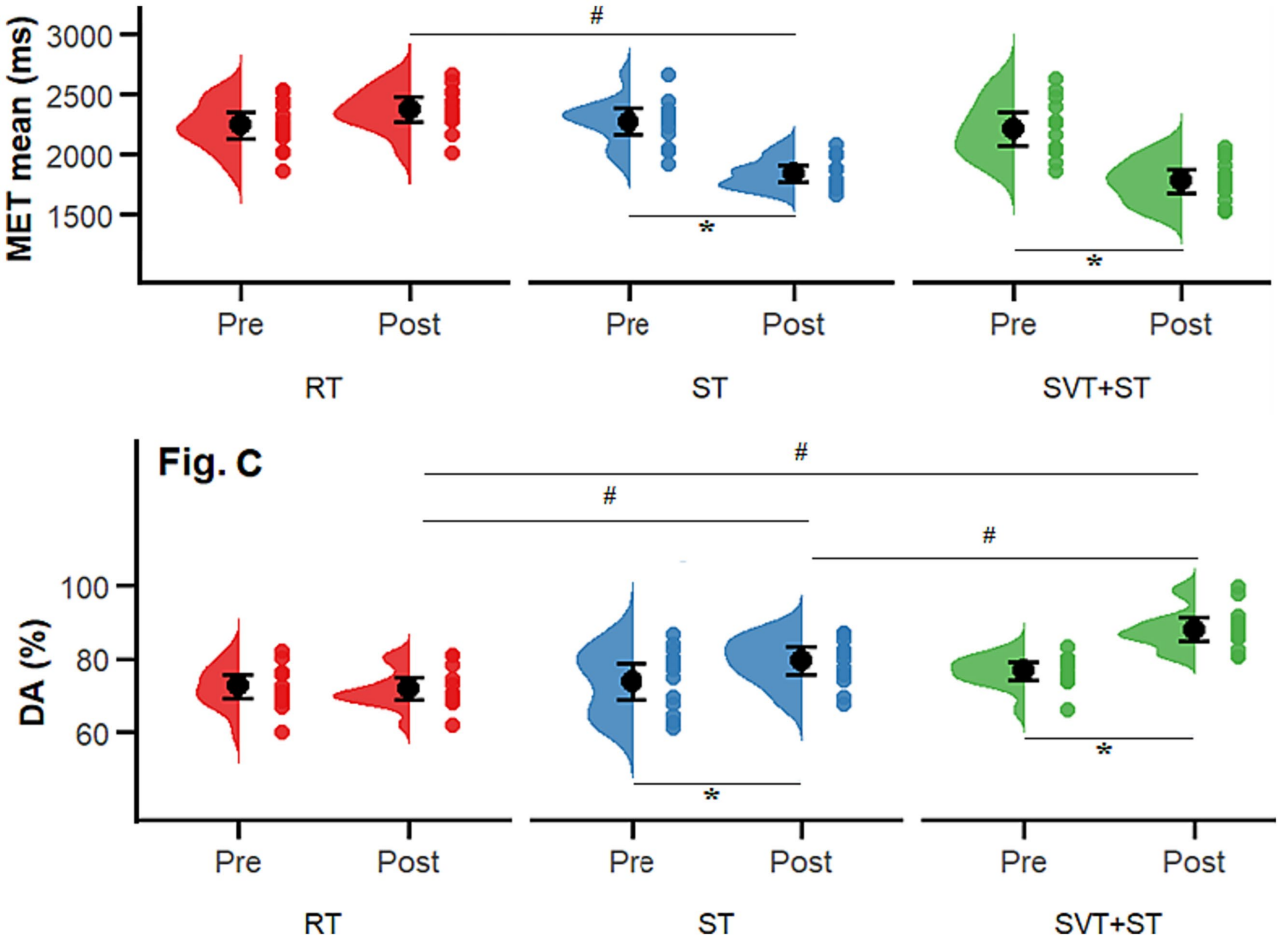
Comparison of 3D tactical animation decision-making results before and after the 8-week intervention across the SVT + ST, ST, and RT groups. **(A)** completion time (s); **(B)** error count (n). Distributions and individual data points are shown for Pre and Post; black dots with error bars indicate the group mean and its uncertainty. * indicates a significant within-group Pre–Post difference (*p* < 0.05); # indicates a significant between-group difference (*p* < 0.05).

A total of 42 participants completed the entire intervention, and all data were included in the final statistical analysis. The Shapiro–Wilk test confirmed that all outcome variables followed a normal distribution (*p* > 0.05). No significant between-group differences were observed in demographic characteristics (age, height, weight; see Table 1) or in baseline outcome measures: Completion Time (*p* = 0.358), Error Count (*p* = 0.851), Completion Time (*p* = 0.975), Left–Right Difference (*p* = 0.898), Decision Time (DT, *p* = 0.898), Motor Execution Time (MET, *p* = 0.330), Decision Accuracy (DA, *p* = 0.360), and the Cognitive–Motor Efficiency Index (CMEI, *p* = 0.140). Accordingly, a mixed-design analysis of variance (ANOVA) with time (TIME) and group (GROUP) as main factors was conducted to examine changes in coordination, agility, and decision-making indices across the three groups. Descriptive statistics for pre- and post-test measures are presented in Table 2, and interaction effects between time and group (TIME × GROUP) are illustrated in Figures 2–4.

**Table 2:** Descriptive statistics of pre- and post-training results for the SVT + ST, ST, and RT groups.

Variable		SVT+ST (*n* = 14)		ST (*n* = 14)		RT (*n* = 14)	
		Pre	Post	Pre	Post	Pre	Post
Coordination	Completion time (s)	12.1 ± 1.08	10.44 ± 1.0	12.07 ± 0.68	11.35 ± 0.98	12.56 ± 1.20	12.17 ± 1.03
	Error count (*n*)	3.36 ± 1.34	1.21 ± 1.12	3.36 ± 1.50	2.29 ± 0.83	3.64 ± 1.74	3.36 ± 1.22
Agility	Completion time (s)	3.47 ± 0.50	2.11 ± 0.30	3.48 ± 0.45	2.71 ± 0.35	3.51 ± 0.52	3.44 ± 0.47
	Left–right difference (%)	7.26 ± 1.28	4.99 ± 1.27	7.36 ± 0.96	5.15 ± 1.02	7.17 ± 0.94	6.99 ± 0.98
Decision-making capacity	DT mean (ms)	575.92 ± 31.91	450.47 ± 58.02	589.12 ± 56.44	495.69 ± 58.83	575.08 ± 60.51	560.59 ± 58.44
	MET mean (ms)	2259.27 ± 218.04	1777.38 ± 188.83	2386.51 ± 244.01	1888.45 ± 128.29	2305.85 ± 231.6	2322.5 ± 183.11
	DA%	75.96 ± 5.65	86.7 ± 4.55	72.69 ± 7.79	78.86 ± 7.44	73 ± 5.27	73.49 ± 4.99
	CMEI	26.89 ± 2.18	39.18 ± 3.79	24.66 ± 3.93	33.07 ± 2.61	25.45 ± 2.29	25.59 ± 2.38

Data presented as mean ± standard deviation; DT, decision time; MET, movement execution time; DA, decision accuracy; CMEI, cognitive-motor efficiency index.

### 3.1 Coordination

As illustrated in Figure 2, results from the Harre Circuit Test indicated significant improvements in coordination performance following the intervention. For completion time, significant main effects were observed for time [*F* (1, 39) = 30.372, *p* < 0.01, ηp^2^ = 0.438 large], group [*F* (2, 39) = 5.539, *p* = 0.006, ηp^2^ = 0.233 large], and their interaction [*F* (2, 39) = 5.084, *p* = 0.011, ηp^2^ = 0.207 large]. For error count, the main effects of time [*F* (1, 39) = 87.431, *p* < 0.01, ηp^2^ = 0.692 large], group [*F* (2, 39) = 3.279, *p* = 0.048, ηp^2^ = 0.144 large], and their interaction [*F* (2, 39) = 18.608, *p* < 0.01, ηp^2^ = 0.488 large] were also statistically significant. *Post hoc* comparisons revealed that the SVT + ST group completed the test significantly faster than both the ST and RT groups (*p* = 0.013, Cohen’s d = 0.763 large) and committed significantly fewer errors than the RT group (*p* < 0.01, d = 4.009 large).

### 3.2 505 change-of-direction speed test

As illustrated in Figure 3, the 505 Change-of-Direction Speed Test results showed significant improvements in movement efficiency across groups following training. For completion time, there were significant main effects of time [*F* (1, 39) = 264.87, *p* < 0.001, ηp^2^ = 0.87 large], group [*F* (2, 39) = 9.93, *p* < 0.001, ηp^2^ = 0.337 large], and their interaction [*F* (2, 39) = 68.72, *p* < 0.001, ηp^2^ = 0.778 large]. For the left–right difference index, significant main effects were also found for time [*F* (1, 39) = 119.53, *p* < 0.001, ηp^2^ = 0.75 large], group [*F* (2, 39) = 3.91, *p* = 0.028, ηp^2^ = 0.17 large], and their interaction [*F* (2, 39) = 14.41, *p* < 0.001, ηp^2^ = 0.43 large]. Post hoc analyses indicated that the SVT + ST group completed the test significantly faster than both the ST and RT groups (*p* < 0.001, Cohen’s d = 3.943 large) and demonstrated markedly reduced left–right asymmetry compared with the RT group (*p* < 0.001, d = 2.074 large).

### 3.3 Decision-making

As illustrated in Figure 4, the 3D tactical-animation test results revealed significant effects of the intervention on athletes’ perceptual–cognitive performance. For mean decision time (DT), significant main effects were found for time [*F* (1, 39) = 119.53, *p* < 0.001, ηp^2^ = 0.75 large], group [*F* (2, 39) = 4.652, *p* = 0.015, ηp^2^ = 0.193 large], and their interaction [*F* (2, 39) = 14.41, *p* < 0.001, ηp^2^ = 0.43 large]. For mean motor-execution time (MET), the main effects of time [*F* (1, 39) = 60.46, *p* < 0.001, ηp^2^ = 0.61], group [*F* (2, 39) = 7.72, *p* = 0.002, ηp^2^ = 0.28 large], and their interaction [*F* (2, 39) = 18.37, *p* < 0.001, ηp^2^ = 0.49 large] were also significant. Post-hoc comparisons showed that the SVT + ST group demonstrated significantly faster decision times than both the ST and RT groups (*p* < 0.001, Cohen’s d = 1.909 large) and faster motor-execution times than the RT group (*p* < 0.001, d = 2.102 large).

The 3D tactical-animation test revealed significant improvements in both decision accuracy and cognitive–motor efficiency following the intervention. For decision accuracy (DA%), significant main effects were found for time [*F* (1, 39) = 78.46, *p* < 0.001, ηp^2^ = 0.67 large], group [*F* (2, 39) = 8.51, *p* = 0.001, ηp^2^ = 0.30 large], and their interaction [*F* (2, 39) = 18.13, *p* < 0.001, ηp^2^ = 0.48 large]. For the cognitive–motor efficiency index (CMEI), the main effects of time [*F* (1, 39) = 186.46, *p* < 0.001, ηp^2^ = 0.83], group [*F* (2, 39) = 33.10, *p* < 0.001, ηp^2^ = 0.63 large], and their interaction [*F* (2, 39) = 72.46, *p* < 0.001, ηp^2^ = 0.79 large] were also statistically significant. Post-hoc comparisons indicated that the SVT + ST group achieved significantly higher decision accuracy than both the ST and RT groups (*p* < 0.001, Cohen’s d = 2.341 large) and significantly greater cognitive–motor efficiency compared with both the ST and RT groups (*p* < 0.001, d = 3.221 large).

## 4 Discussion

This study examined the combined effects of SVTand on coordination, 505 change-of-direction speed, and decision-making in collegiate basketball players. The results showed that participants in the SVT + ST group outperformed those in the ST and RT groups across all performance domains. Specifically, the SVT + ST group demonstrated faster completion times in coordination and 505 change-of-direction speed tasks, shorter reaction and motor-execution times in decision-making assessments, and higher decision accuracy and cognitive–motor efficiency. Collectively, these findings suggest that integrating SVT into basketball-specific training yields substantial benefits for perceptual–motor performance, supporting the concept of perception–action integration in sport-specific contexts. The primary causal inference regarding SVT is based on the dose-matched comparison between SVT + ST and ST, where training content and exposure were identical and only the visual perturbation differed. It is important to emphasize that, although the present study employed standardized tests that were designed to approximate basketball game demands, whether the observed improvements generalize to competitive match performance remains uncertain. Competitive basketball imposes additional constraints, including opponent pressure, tactical variability, and fluctuations in physical and mental fatigue, which cannot be fully represented in controlled assessments of coordination, change-of-direction speed, or simulated decision-making tasks. Accordingly, the findings should be interpreted as evidence that SVT improves test-based perception-action and cognition-action performance under standardized conditions, rather than as a demonstration of direct improvements in real match performance.

The SVT ± ST group demonstrated greater improvements in coordination than the ST and RT groups, primarily evidenced by shorter completion times on the coordination test. By intermittently disrupting visual input, stroboscopic vision requires participants to sustain stable motor execution under conditions of incomplete visual information, thereby enhancing visuomotor integration and promoting the use of predictive control (Jothi et al., 2025; Sudesan et al., 2023). This account closely matches the task demands of basketball, in which dribbling, receiving/passing, and shot preparation depend on rapid cue extraction and hand–eye coordination under substantial time pressure (Güner et al., 2024; Dako et al., 2024). When visual sampling is interrupted, the central nervous system may rely more heavily on feedforward control and internal prediction to preserve movement quality; consequently, once normal vision is restored, training benefits may be expressed as more efficient motor organization and execution (Pekna et al., 2012). Previous research mainly comprises two lines of evidence: acute exposure studies and periodized training interventions. In a controlled design, Ellison et al. (2020) implemented SVT and assessed hand-eye coordination using an electronic reaction light system. Their findings showed improvements in relevant response measures after SVT, indicating that visuomotor integration may be trainable. Wilkins et al. (2018) reported improved perceptual response performance in elite adolescent football goalkeepers, consistent with the notion that manipulating visual information availability may facilitate more efficient visual processing and motor control strategies. Importantly, most of this evidence comes from other open-skill sports or generic tasks, and the movement constraints do not fully correspond to key basketball situations. Basketball imposes high demands on visual sampling and rapid movement adjustment under ongoing opposition, and is characterized by strong continuous closed-loop control requirements (Newell and Rovegno, 2021). Against this backdrop, embedding SVT within sport-specific training and observing larger improvements on coordination tests may reflect increased training demands on perception-action coupling while maintaining task representativeness (Carrolla et al., 2021). Periodized evidence also supports the rationale for progressive load scheduling. For example, Hülsdünker et al. (2019) reported in elite badminton athletes that SVT was associated with improvements in visual function and sport-specific visuomotor performance. Moreover, a series of studies by Hülsdünker et al. (2021a) focusing on MSSE suggests that visuomotor reaction speed may exhibit both short-term and long-term adaptations. Taken together, the coordination gains observed following the phase-based progressive training in the present study may relate to more efficient cue use, enhanced predictive control, and improved movement stability. At the same time, SVT effects are task-specific and context-dependent (Mitroff et al., 2013). Some findings indicate that changes in dribbling performance under restricted visual feedback may be moderated by factors such as skill level (Luo et al., 2025). Accordingly, the mechanistic account offered here remains an inference consistent with the behavioral outcomes, and future work should integrate eye-tracking and neurophysiological measures to test the proposed processes more directly. SVT was shown to significantly improve change-of-direction speed and reactive agility in collegiate basketball players while simultaneously reducing, to some extent, asymmetry between left- and right-side responses. These outcomes likely result from SVT accelerating the transformation of visual input into motor output, thereby enhancing the overall efficiency of reaction processes. In a study involving youth volleyball players, Zwierko M. et al. (2023) found that after 6 weeks of SVT intervention, participants demonstrated markedly greater visually induced reactive agility than those in the control group. Similarly, Hülsdünker et al. (2021a) reported that 10 weeks of SVT reduced visuomotor reaction time in elite youth badminton players from 251 ms to 238 ms, corresponding to a 5.2% decrease (d = 0.63), and the improvement was largely retained after 6 weeks (241 ms; 4.0% below baseline; d = 0.50), indicating a sustained enhancement in visuomotor reaction speed. In terms of bilateral agility balance, athletes in the SVT group exhibited reduced asymmetry in change-of-direction responses, reflecting more symmetrical agility performance. This improvement may stem from SVT’s simultaneous stimulation of bilateral visual pathways and motor control systems, which enhances neuromuscular coordination, particularly on the non-dominant side. Supporting this interpretation, Lu et al. (2025) reported that collegiate male soccer players who underwent balance training combined with SVT achieved significant improvements in both dominant- and non-dominant-leg stability, alongside notable reductions in inter-limb functional differences. Regarding bilateral performance, we observed a reduction in left–right asymmetry in change-of-direction (COD) responses in the SVT group, indicating a shift toward more symmetrical COD performance. This finding may be practically meaningful in basketball, where frequent stop–start actions, defensive slides, and cutting to both sides can foster side-preferential strategies; accordingly, stable limb-to-limb differences are often seen with prolonged training and competitive exposure (Shimokochi et al., 2013; Stacoff et al., 1996). One plausible explanation is that intermittent visual disruption under stroboscopic vision reduces the continuity of visual input, making athletes less reliant on online visual corrections during rapid foot placement, braking, and re-acceleration. In turn, they may rely more on feedforward control and proprioceptive monitoring to stabilize trunk and lower-limb mechanics (Elliott and Bennett, 2021; Zennou-Azogui et al., 1996). For the non-dominant side, a control strategy that emphasizes internal prediction and sensory feedback may be especially conducive to improving movement stability and timing consistency, thereby narrowing left–right discrepancies (Sullivan and Rodewald, 2012; Das et al., 2025). In addition, our SVT ± ST training embedded visual perturbation within sport-specific drills that combined bi-directional COD with response selection. This likely provided the non-dominant side with more frequent and higher-quality exposure to decision-coupled deceleration–acceleration demands, facilitating a “catch-up” adaptation and reducing inter-limb asymmetry.

Athletes who underwent SVT demonstrated notable improvements in decision reaction time, judgment accuracy, and motor execution speed, suggesting that SVT exerts a substantial positive influence on perceptual–cognitive decision-making. These enhancements likely result from increased neural processing efficiency within the perception–action pathway (Li et al., 2024). On one hand, SVT has been shown to facilitate neural transmission along the dorsal visual stream—particularly through the magnocellular (M) pathway—by accelerating motion-related visual processing and potentially inducing neurophysiological changes such as alpha-wave phase resetting. Supporting this mechanism, Zwierko T. et al. (2023) found that after 6 weeks of SVT, handball players exhibited significantly reduced P100 latency in visual evoked potentials, particularly in the dominant eye and binocular peripheral pathways, indicating faster early-stage visual processing. Enhanced perceptual extraction enables athletes to identify and prioritize key visual cues more efficiently in complex, dynamic environments, thereby reducing decision reaction time (Huang et al., 2018). On the other hand, SVT appears to strengthen predictive and attentional control mechanisms within the brain, which together improve judgment accuracy and motor efficiency. Previous work indicates that training under intermittent (stroboscopic) vision can enhance visual information encoding and short-term retention, thereby improving the ability to use brief, transient cues when visual sampling is constrained (Appelbaum et al., 2011). Building on this framework, the concurrent improvements in decision speed and accuracy observed in the present study may reflect more efficient extraction of task-relevant cues within shortened viewing windows and better maintenance of those cues during occlusion gaps, reducing reliance on late, online corrections during action execution (Jothi et al., 2025; Ren et al., 2025). In addition, intermittent vision likely increases attentional engagement and supports motion extrapolation/anticipatory timing processes, which can facilitate earlier and more consistent response initiation under time pressure—consistent with the observed gains in reaction-based outcomes and perceptual–motor efficiency (Ballester et al., 2017).

Although the findings of this study are broadly consistent with previous research supporting the performance-enhancing effects of SVT, certain discrepancies remain. For example, Palmer et al. (2022) reported that a four-week SVT program did not significantly improve dribbling or ball-control ability in youth soccer players. The authors suggested that the complex technical and tactical demands of soccer may require a longer intervention period or greater contextual integration of SVT into game-like scenarios to produce measurable benefits. In contrast, the present study observed significant improvements in coordination, change-of-direction speed, and decision-making among basketball players, indicating that the effectiveness of SVT may depend on sport-specific skill structures and movement characteristics. Additionally, previous research has emphasized different performance domains. Zwierko M. et al. (2023) found that SVT primarily enhances visuomotor responsiveness closely linked to motor execution, whereas its influence on purely sensory functions, such as peripheral visual acuity, appears limited. Similarly, Appelbaum et al. (2011) demonstrated that SVT improves information-processing efficiency in central vision but has a weaker impact on peripheral perceptual sensitivity. The current findings, which focus on coordination, 505 change-of-direction speed, and decision-making—abilities highly dependent on perception–action coupling—suggest that SVT may exert its strongest effects in performance areas where visual perception and motor execution are tightly integrated. Nevertheless, some studies have reported mild adverse reactions during SVT training. For instance, Vasile and Stănescu (2024) noted slight dizziness and visual fatigue among participants in a climbing-based SVT program. Although no such effects were observed in the present research, these reports highlight the necessity of monitoring individual tolerance levels and carefully adjusting strobe frequency, duration, and progression to ensure safety and long-term feasibility in applied training contexts.

## 5 Limitations and future research

Although this study demonstrated that SVT combined with basketball-specific training can enhance coordination, agility, and decision-making in collegiate basketball players, several limitations should be acknowledged. First, the relatively small sample size and the inclusion of only male athletes from a single university limit the generalizability of the findings. Future research should expand the participant pool to include athletes of different genders, competitive levels, and regional backgrounds to improve external validity and representativeness. Second, the intervention period of 8 weeks and the absence of follow-up assessments restrict the ability to determine the persistence and cumulative effects of SVT. Future studies should extend the duration of training and incorporate medium- and long-term follow-ups to systematically evaluate the sustainability and potential dose–response relationship of SVT-induced adaptations. Third, the outcome variables in this study were primarily behavioral—coordination, 505 change-of-direction speed, and decision-making—without inclusion of neurophysiological or biomechanical measures. This limits the understanding of the underlying mechanisms driving the observed improvements. Incorporating neural imaging, electrophysiological, or eye-tracking indicators in future work could help elucidate how SVT affects the perception–action coupling process at the neural level. Fourth, participants in the present study represent a collegiate performance level; therefore, the findings may not generalize to higher-performance or professional players. Following the participant classification framework proposed by McKay et al. (2022), future studies should explicitly report training/performance caliber and test SVT effects across multiple competitive tiers. In addition, we did not directly quantify or control physical and mental fatigue status (e.g., sleep/wellness, accumulated training stress, or pre-testing cognitive load), which may influence both perceptual–cognitive and skilled performance and contribute to inter-individual variability in training responses. Given evidence that mental fatigue can impair sport-related performance, future SVT studies should incorporate standardized fatigue monitoring (e.g., wellness/sleep questionnaires, session-RPE and training-load metrics, and mental-fatigue ratings) and, where possible, standardize recovery and testing timing relative to training load. Finally, whether the improvements in decision-making performance observed under simulated conditions can transfer effectively to real-game scenarios remains uncertain. Subsequent research should adopt ecologically valid performance metrics and on-court behavioral analyses to verify the transferability of SVT effects to competitive play.

## 6 Conclusion

Integrating SVT into conventional basketball-specific programs significantly enhances coordination, 505 change-of-direction speed, and decision-making in collegiate basketball players. Compared with athletes who received only sport-specific training, those in the SVT group achieved greater improvements across these domains, indicating the additional benefits of incorporating perceptual-interference methods. By introducing intermittent visual occlusion to increase perceptual–cognitive load, this combined training approach effectively reinforces athletes’ perception–action integration, enabling superior performance in complex and dynamic environments. Overall, the integration of SVT with sport-specific training demonstrates positive effects on both physical and cognitive aspects of athletic ability, offering theoretical insight and practical guidance for optimizing training program design. Future studies should further investigate the long-term efficacy of SVT and its potential integration with other training modalities to achieve more comprehensive performance enhancement.

## Data Availability

The original contributions presented in the study are included in the article/Supplementary material, further inquiries can be directed to the corresponding authors.
