# Supplementary material for: The effects of stroboscopic visual training on coordination, change-of-direction, and decision-making performance in collegiate basketball players
Source: Front Psychol. 2026 Feb 18;17:1750065. doi: 10.3389/fpsyg.2026.1750065 (PMC12956791; doi:10.3389/fpsyg.2026.1750065)
Supplement: Supplementary file 1 [file Presentation_1.pdf]

Appendix 1. Typical weekly training exposure and the 8-week basketball-specific program

Table A1. Weekly training exposure

Variable	Value
Training frequency	3 sessions/week (Mon/Wed/Fri)
Session duration	40 min/session
Weekly total volume	120 min/week (2.0 h/week)
Basketball activity exposure	20 min/session → 60 min/week (1.0 h/week)
Physical conditioning exposure	20 min/session → 60 min/week (1.0 h/week)

Footnote A1. Time-category rule (applied to all sessions). Each 40-min session was a priori reported as 20 min basketball activity + 20 min physical conditioning. "Basketball activity" refers to ball-involved skill/tactical execution segments (e.g., ball-handling/passing, shooting, moving/catch-and-shoot, and basketball-situated decision execution). "Physical conditioning" refers to segments primarily intended to induce physiological load, including warm-up and cool-down, plus conditioning-oriented, higher-tempo movement demands embedded in drill blocks (e.g., repeated COD patterns, shuttle components, and small-sided competitive bouts).

Table A2. Typical weekly training schedule

Reporting allocation per session (fixed): basketball activity = 20 min; physical conditioning = 20 min (see Footnote A1).

Day	Session	Session structure (organisation)	Key prescription (sets × reps/time @ intensity)	Work: Rest	Target intensity	Duration
Mon	Session 1	Warm-up (≈8) → Block A: ball-handling & passing (≈10) → Block B: shooting (≈10) → Block C: peripheral cueing + simple cue–response decisions (≈8) → Cool-down (≈4)	Ball-handling: 3 × 30 s/hand; passing: 3 × (10 chest + 10 bounce + 10 one-hand); five-spot shooting: 5 spots × 6–10 shots; peripheral cues: ≥5 responses/set × 3 sets; cue-based actions (shoot/pass/stop): ≥8 correct responses/set × 2 sets	W1–4: 30s:30s; W5–8: 45s:45s	RPE 5–7	40
Wed	Session 2	Warm-up → Block A: dynamic dribbling + COD patterns → Block B: moving/catch-and-shoot → Block C: three-choice decisions + 2v1/3v2 transitions → Cool-down	COD dribble patterns: 4–6 patterns × 2–3 reps; moving shots: ≥20 shots/session; three-choice decisions (e.g., red/green/blue): ≥8 correct responses/set × 2–3 sets; 2v1/3v2: 3–4 transitions/set × 2 sets with role rotation	Same as above	RPE 6–8	40
Fri	Session 3	Warm-up → Block A: full-speed obstacle dribbling → Block B: shuttle + shooting → Block C: 2v2/3v3 competitive bouts → Cool-down	Obstacle dribble (5 cones): ≥2 circuits/set × 2 sets; shuttle (10–15 m) + shot: 4–6 shuttles/set × 2 sets; 2v2/3v3: 6–10 bouts × 45–60 s	W5–8 commonly 45s:45s	RPE 6–8	40

Footnote A2. SVT+ST and ST were fully dose-matched (training frequency, session duration, drill content, and work: rest). Only SVT+ST implemented stroboscopic occlusion, progressed across phases to increase visual perturbation while preserving identical movement tasks and total session duration. The RT group completed routine training of equal duration in the same time window.

Table A3. Eight-week basketball-specific program progression

Phase (weeks)	Strobe frequency (Hz)	Duty cycle	Lens mode	Key drill categories	Work/rest & sets	Detailed task prescription
Phase I (Weeks 1–2)	14–16 (individualised start)	50%	Standard strobe; finish with 1–2 min alternating-eye Mode C	Stationary dribbling; spot shooting; peripheral cueing; simple cue–response decisions; near–far focus switching	30s work / 30s rest × 2 sets (≈40 min incl. warm-up/cool-down)	Stationary dribbling: alternate hands (≥30 contacts/set) → add passing (10 chest, 10 bounces, 10 one-hand). Five-point spot shooting: 5 spots × 6–10 shots. Peripheral cueing: respond to random LED cues (≥5 responses/set). Simple decisions: execute shoot/pass/stop within ≤2 s after colour/gesture cue. Near–far focus switches: ≥10 shifts/set.
Phase II (Weeks 3–4)	14–16 (maintained; adjusted as needed)	50%	Standard strobe + Mode C	Moving shots; COD dribble patterns; three-choice decisions; 2v1/3v2 transitions; peripheral reaction	30s work / 30s rest × 2 sets (≈40 min)	Builds on Phase I with greater movement/tactical complexity: COD dribble: 4–6 patterns (e.g., V-cut, L-cut) × 2–3 reps. Moving shooting: catch-and-shoot off cuts (≥20 total shots/session). Tri-choice decisions: respond to 3 cues (e.g., red=shoot, green=drive, blue=pass) (≥8 correct responses/set). 2v1/3v2: 3–4 transition

Phase (weeks)	Strobe frequency (Hz)	Duty cycle	Lens mode	Key drill categories	Work/rest & sets	Detailed task prescription
						plays/set with role rotation.
Phase III (Weeks 5–6)	13–14	50–60%	Mode B/C to increase occlusion load & depth-perception challenge	Full-speed obstacle dribbling; pick-and-roll; shuttle + shooting; dual-task drills; 2v2 competition	45s work / 45s rest × 2 sets (≈40 min)	Shuttle + shooting: 4–6 shuttles (10–15 m) + 1–2 shots/bout (≥4 shots/position). Cone obstacle dribble: 5 cones multidirectional × ≥2 circuits/set. Pick-and-roll reads: 6–10 reps/session with guided coverages. Dual-task: ball control while responding to peripheral cues (≥6 responses/set). 2v2: short competitive bouts emphasising quick reads.
Phase IV (Weeks 7–8)	9–11 (fine-tuned individually)	60–70%	Adaptive frequency; alternating-eye occlusion emphasised	3v3/4v4 scrimmage; fast-break decisions; weak-side cuts; random tactics; rebounding/box-out	45s work / 45s rest × 2–3 sets (game-simulation emphasis)	3v3/4v4 short games with constraints (e.g., shot-clock, no-dribble rules). Fast-break: real-time decision to pass/finish/kick-out (3–5 sequences/set). Weak-side cuts + corner shots under occlusion: 4–5 plays/set. Random coach commands (“switch”, “trap”, “cut back”) requiring ≤1 s

Phase (weeks)	Strobe frequency (Hz)	Duty cycle	Lens mode	Key drill categories	Work/rest & sets	Detailed task prescription
						initiation. Three-man rebounding: box-out + secure (6–10 reps/set).

Footnote A3. SVT+ST and ST sessions were fully dose-matched; stroboscopic occlusion (SVT+ST only) was progressed across phases to increase visual perturbation while preserving identical movement tasks, work:rest structure, and total session duration.

Abbreviations

COD, change of direction; Hz, hertz; RPE, rating of perceived exertion; SVT, stroboscopic visual training; ST, skill training; RT, routine training.
