# Supplementary material for: The effects of stroboscopic visual training on coordination, change-of-direction, and decision-making performance in collegiate basketball players
Source: Front Psychol. 2026 Feb 18;17:1750065. doi: 10.3389/fpsyg.2026.1750065 (PMC12956791; doi:10.3389/fpsyg.2026.1750065)
Supplement: Supplementary file 2 [file Presentation_2.pdf]

Coordination Test

A modified version of the Harre Circuit Test was used to evaluate participants' whole-body coordination. The original test, developed by German sports scientist Dietrich Harre, was designed to measure athletes' general coordination ability (Harre & Barsch, 1982) and has since been widely applied across various sports disciplines (Ljach & Witkowski, 2010). In this study, the starting action was adapted from the traditional forward roll to a basketball-specific defensive slide and pivot sequence to enhance task specificity while preserving the test's validity as a comprehensive coordination assessment (Scanlan et al., 2014a).

Test Setup.
The testing area measured 6 m × 5 m and included one marker pole, three hurdles (75 cm in height, 2.5 m apart), and a 1 m × 1 m foam mat. The mat was positioned 1 m from the start line, the marker pole was placed 3.5 m away, and hurdles were numbered 1 to 3 in a counterclockwise direction. All trials were performed on a standard indoor basketball surface to ensure uniform traction (Figure 2).

Procedure.
Participants began in a defensive stance behind the start line (feet shoulder-width apart, low center of gravity, hands positioned laterally). Upon the "start" command, they executed a 3 m defensive slide without crossing their feet, followed by a 180° pivot turn (Spiteri et al., 2014) and sprint toward the marker pole. After passing the pole from the left, they changed direction toward the first hurdle, jumped over it, and immediately ducked underneath. This "jump-over–crawl-under" sequence was repeated for the remaining two hurdles. Participants then leaped over the mat and sprinted through the finish line. Each participant completed three trials, with at least five minutes of rest between attempts.

Technical Standards.
During the defensive slide, feet could not cross, posture had to remain low, and hands were required to stay within the frontal plane. The pivot turn had to be complete, with no additional adjustment steps, and contact with the pole or hurdles was prohibited. When jumping, both feet had to leave and land simultaneously; during the crawl, hips had to remain below hurdle height. A photoelectric timing system (Brower Timing Systems, Salt Lake City, UT, USA) recorded completion time with 0.01 s precision.

Error Evaluation.
Two independent evaluators observed each trial and recorded errors, assigning one point per infraction. Error types included: (1) crossing feet or raising the torso; (2) incomplete or multi-step turns; (3) touching or knocking over a pole or hurdle; (4)

one-legged jumping or landing; (5) raising hips above hurdle height; (6) stepping on the mat edge; and (7) incorrect sequence execution. Trials with three or more errors were deemed invalid and repeated after a five-minute rest. All evaluators underwent standardized training through video and live demonstrations to ensure scoring consistency.

Outcome Measures and Reliability.

The primary outcome variables were completion time (s) and the number of technical errors. The best valid trial (fastest completion time) was used for analysis. In a pilot test–retest with 20 participants conducted one week apart, reliability for completion time was ICC = 0.89 (95% CI: 0.76–0.95), and for error count ICC = 0.86 (95% CI: 0.70–0.94). Inter-rater reliability was Kappa = 0.88 (95% CI: 0.76–0.94), demonstrating high evaluator consistency. The modified Harre Circuit Test retained the original version's capacity to assess multidirectional movement, body control, and spatial orientation (Ljach & Witkowski, 2010), while the inclusion of basketball-specific defensive elements enhanced its ecological validity and sport relevance.

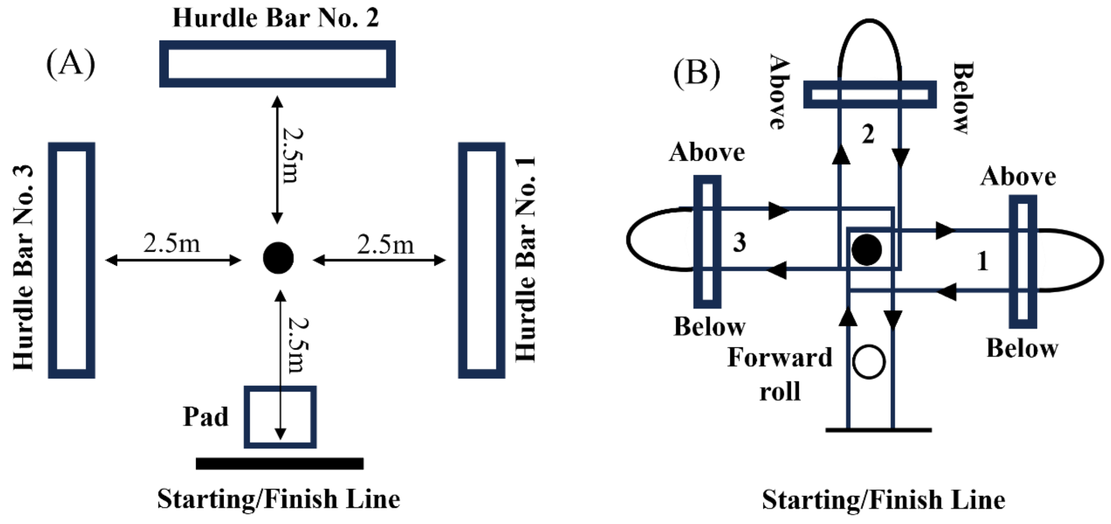

Figure 2. *Harley Test*
