# Supplementary material for: The effects of stroboscopic visual training on coordination, change-of-direction, and decision-making performance in collegiate basketball players
Source: Front Psychol. 2026 Feb 18;17:1750065. doi: 10.3389/fpsyg.2026.1750065 (PMC12956791; doi:10.3389/fpsyg.2026.1750065)
Supplement: Supplementary file 3 [file Presentation_3.pdf]

505 Change-of-Direction Speed Test

The 505 Change-of-Direction Speed Test was employed to assess participants' ability to decelerate, perform a 180° change of direction, and reaccelerate at high speed (Draper & Lancaster, 1985). Standardized by the Australian Institute of Sport (AIS), this test has been widely applied in basketball-related research and demonstrates excellent test–retest reliability (ICC = 0.94–0.96) and criterion validity (Nimphius et al., 2018).

Test Setup.

The test was conducted on a 15 m straight sprint track with clear ground markings. A photoelectric timing gate (Brower Timing Systems, Salt Lake City, UT, USA) was positioned 5 m before the turning line to initiate and stop timing, while a 5 cm wide tape marker was affixed at the turning line to indicate the change-of-direction point (Figure 3).

Prior to baseline testing, each participant's dominant leg was determined using three complementary methods:

1. **Single-leg horizontal jump test** — three attempts per side, with the leg producing the longer distance identified as dominant;
2. **Simulated kicking-leg preference** — observation of preferred leg during a mock kicking motion;
3. **Self-reported daily activity preference.**

When all three indicators were consistent, dominance was confirmed; when inconsistent, the jump test result was prioritized. The overall consistency rate for dominance determination among participants was 95.2% (40/42).

Procedure.

Participants began with a flying start from a point 15 m behind the turning line. Timing began as the torso crossed the photocell gate located 5 m before the turning line and stopped when the participant returned through the same gate. Each trial involved sprinting to the turning line, making full-foot contact or crossing the line, executing a 180° pivot turn (to the left or right depending on test side), and reaccelerating back through the gate. The effective timed distance was therefore 10 m (5 m approach + 5 m return). Each participant completed three trials per side, alternating sides, with a minimum of 3 minutes of rest between trials to minimize fatigue. Trials were repeated if premature deceleration or insufficient reacceleration was observed.

Evaluation Metrics.

Two primary outcomes were recorded: **completion time (s)** and **asymmetry index**

(AI%). Completion times were reported separately for the dominant and non-dominant sides, as well as the bilateral mean. The asymmetry index, representing interlimb performance balance, was calculated as:

$$AI = \frac{|T_{dom} - T_{non}|}{\frac{T_{dom} + T_{non}}{2}} \times 100$$

Note: *T_{dom} = completion time for the dominant leg (s); T_{non} = completion time for the non-dominant leg (s)*

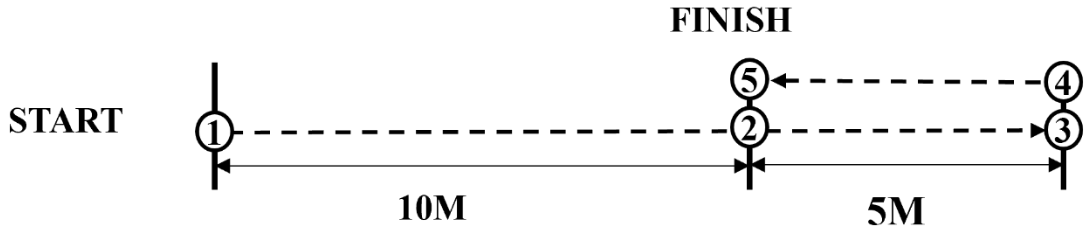

Figure 3. *505 Test*

Reliability.

Preliminary test–retest analysis demonstrated high reliability: ICC = 0.93 (95% CI: 0.85–0.97) for the dominant leg, ICC = 0.91 (95% CI: 0.81–0.96) for the non-dominant leg, and ICC = 0.87 (95% CI: 0.72–0.94) for the asymmetry index. The minimal detectable change (MDC_{95}) was 0.11 s.
