# Supplementary material for: The effects of stroboscopic visual training on coordination, change-of-direction, and decision-making performance in collegiate basketball players
Source: Front Psychol. 2026 Feb 18;17:1750065. doi: 10.3389/fpsyg.2026.1750065 (PMC12956791; doi:10.3389/fpsyg.2026.1750065)
Supplement: Supplementary file 4 [file Presentation_4.pdf]

Decision-Making Test

System Overview

A customized decision-making assessment system integrating three-dimensional (3D) tactical animations with real-action execution was used to evaluate athletes' perception–judgment–action performance under simulated game conditions. Developed with the Unity engine, the system rendered high-fidelity basketball scenarios that combined visual–cognitive demands with physical responses, thereby providing an ecologically valid measure of sport-specific decision making and implementation ability.

Development and Validation

An expert panel comprising five national-level basketball coaches and three collegiate head coaches identified 30 representative tactical scenarios based on analyses of Chinese Basketball Association (CBA) and National Collegiate Athletic Association (NCAA) game footage. The set included 10 fast-break 2v1, 10 pick-and-roll 3v2, and 10 corner-spacing situations.
Using a three-round Delphi process, the experts reached consensus on the optimal decision for each scenario, defined as ≥87.5% agreement (≥7 of 8 experts). The final consensus rate was 92.5%, confirming the scenarios' representativeness and reliability.

Content validity was strong (CVI = 0.91), with mean expert ratings of 4.6/5.0 for realism and relevance. Criterion validity testing (n = 30) revealed significant correlations between test performance and players' assist-to-turnover ratios ($r = 0.58$, $p < 0.01$) as well as coach-rated basketball IQ ($r = 0.64$, $p < 0.01$).

Testing Environment

Participants stood 3 m from a 65-inch LED screen (Samsung UN65RU7100, 3840 × 2160 px) positioned at eye level. The setup included a 1 m × 1 m passing target (1.5 m high, 5 m away), a regulation three-point line (6.75 m), and cones placed along a 5 m sprint path.
Stimuli presentation and response capture were managed through E-Prime 3.0 (Psychology Software Tools, Inc.). Responses were color-coded: green = pass, blue = shoot, red = drive. A high-speed camera (Sony RX100 VII, 240 fps) recorded all motor-execution phases for subsequent time analysis.

Procedure

Thirty randomized 3D tactical clips (3–5 s each) were displayed, freezing for 0.5 s at the decision frame. Participants had an 800 ms reaction window to select the

appropriate option via key press.

- Decision Time (DT, ms): measured from the freeze frame to key press; correct responses matched expert consensus.
- Following the on-screen feedback color, participants performed the associated real-action task:
    - Pass: two-hand chest pass to the 1 m × 1 m target 5 m away.
    - Shoot: standard three-point shot from beyond the arc (6.75 m).
    - Drive: dribble 5 m to the cone, touch it, and return.

High-speed video was used to calculate Motor-Execution Time (MET, ms)—the interval from visual feedback onset to task completion (ball release for pass/shoot, or cone-touch return for drive).

Three practice trials preceded testing. The 30 clips were presented in random order with 5 s between trials and a 1-min rest every 10 trials. Total duration was approximately 12 min. Environmental conditions were strictly controlled (noise < 40 dB, temperature 22–24 °C, illumination 500–750 lux).

Outcome Measures

- Mean Decision Time (DT, ms): latency from stimulus freeze to response, reflecting decision speed.
- Mean Motor-Execution Time (MET, ms): time from visual cue to action completion, indicating motor efficiency.
- Decision Accuracy (DA %): (correct decisions ÷ total) × 100, representing decision quality.
- Cognitive–Motor Efficiency Index (CMEI):

$$CMEI\ （(\%/ms）= \frac{DA\%}{DT + MET}$$

- CMEI expresses correct-decision percentage per millisecond (%/ms); higher values denote greater integrated efficiency of decision making and motor execution.

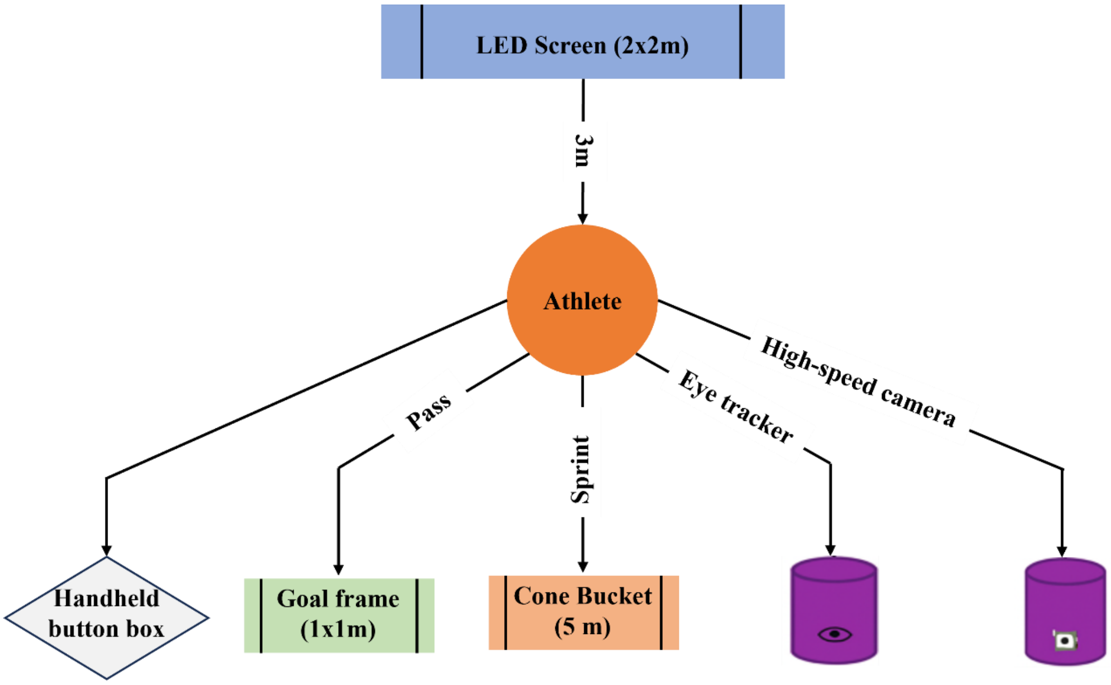

Figure 3. Decision-making ability test setup

Reliability Testing

Test–retest reliability (*n* = 20, one-week interval) was high: DT (ICC = 0.87, 95% CI = 0.71–0.94), MET (ICC = 0.89, 95% CI = 0.75–0.95), DA% (ICC = 0.84, 95% CI = 0.66–0.93), and CMEI (ICC = 0.86, 95% CI = 0.69–0.94). Internal consistency was acceptable (Cronbach's α = 0.82). No significant learning effect was detected between practice and initial trials ($p = 0.78$), confirming stability after standardized familiarization.
